# Closed genome sequences of clinical *Listeria monocytogenes* isolates from salmon-associated listeriosis outbreaks

**DOI:** 10.1128/mra.01311-25

**Published:** 2026-01-14

**Authors:** Sabrina Wamp, Sven Halbedel

**Affiliations:** 1 FG11 Division of Enteropathogenic Bacteria and Legionella, Consultant Laboratory for Listeria, Robert Koch Institute 9222, Wernigerode, Germany; 2 Institute for Medical Microbiology and Hospital Hygiene, Otto von Guericke University Magdeburg, Magdeburg, Germany; University of Manitoba, Winnipeg, Manitoba, Canada

**Keywords:** invasive listeriosis, molecular surveillance, whole-genome sequencing, reference genome

## Abstract

Here, we present the closed genome sequences of nine representative clinical *Listeria monocytogenes* isolates from nine invasive listeriosis outbreaks in Germany that were associated with contaminated salmon products. More than half of the genomes contained two or more intact prophages.

## ANNOUNCEMENT

Listeriosis is a severe foodborne infection. Salmon products contaminated with the bacterium *Listeria monocytogenes* are one of the major causes of listeriosis in Germany (1), where 24 salmon-associated outbreak clusters have been detected in 2010–2022 based on core genome multi-locus sequence typing (cgMLST) (1–3). Here, we present nine closed genome sequences of representative *L. monocytogenes* isolates from nine selected salmon-related outbreaks that belong to different sequence types according to multi-locus sequence typing (MLST). Five of these outbreak clones (Omikron 1, Rho3, Ypsilon6, My2, and Omega5; Table 1) were still causing listeriosis cases in Germany in 2025.

**TABLE 1:** Key characteristics of the genomes sequenced in this study

Isolate ID	17-01049	18-00258	18-04417	19-00348	19-00663	19-03740	19-06323	20-01921	21-05647
Outbreak	Omikron 1	Rho3	Sigma5	Ypsilon6	My2	Omega5	Chi1a	Rho8	Zeta1
Source	Clinical isolate	Clinical isolate	Clinical isolate	Clinical isolate	Clinical isolate	Clinical isolate	Clinical isolate	Clinical isolate	Clinical isolate
Year of isolation	2017	2018	2018	2019	2019	2019	2019	2020	2021
GenBank accession no.	CP169661	CP169663	CP169667	CP169668	CP169669	CP169670	CP169673	CP169677	CP169684
ENA accession no.	SAMEA6800785	SAMEA4920508	SAMEA10017322	SAMEA10018405	SAMEA10018406	SAMEA10017342	SAMEA10017351	SAMEA7540781	SAMEA114338563
Long-read sequencing method (Flow cell/Kit)	MinION, FLOMIN 106/SQK-RBK004	MinION, FLOMIN 114/SQK-NBD114.24	MinION, FLOMIN 106/SQK-RBK004	MinION, FLOMIN 114/SQK-NBD114.24	MinION, FLOMIN 106/SQK-RBK004	MinION, FLOMIN 106/SQK-RBK004	MinION, FLOMIN 114/SQK-NBD114.24	MinION, FLOMIN 114/SQK-NBD114.24	MinION, FLOMIN 114/SQK-NBD114.24
Short-read sequencing method	Illumina MiSeq (2 × 250 bp)	Illumina MiSeq (2 × 300 bp)	Illumina MiSeq (1 × 150 bp)	Illumina NextSeq (2 × 150 bp)	Illumina MiSeq (2 × 300 bp)	Illumina MiSeq (1 × 150 bp)	Illumina MiSeq (1 × 150 bp)	Illumina NextSeq (2 × 150 bp)	Illumina NextSeq (2 × 150 bp)
Accession no. for long-read raw data	ERX11576224	ERX11576226	ERX11576232	ERX11576233	ERX11576234	ERX11576235	ERX11576238	ERX11576244	ERX11576251
Accession no. for short-read raw data	ERX4076809	ERX2805914	ERX6626653	ERX6682231	ERX6682232	ERX6626673	ERX6626682	ERX4678058	ERX11412512
Number of long reads	200,390	84,872	365,859	76,887	286,115	430,631	84,012	41,413	67,493
N50 long reads	18,094	10,646	15,735	11,341	15,335	14,880	10,192	18,935	12,597
Genome size (bp)	3,068,859	3,028,444	3,045,044	2,911,786	2,962,402	2,954,892	3,045,800	2,915,965	2,919,500
GC content (%)	38.1	38	37.9	38	37.9	38	37.9	38	38
No. of protein-coding genes	3,036	2,979	3,040	2,839	2,906	2,869	2,989	2,853	2,809
No. of rRNA operons	6	6	6	6	6	6	6	6	6
No. of tRNA genes	67	67	67	67	67	67	67	67	67
Pathogenicity island(s)	LIPI-1	LIPI-1	LIPI-1	LIPI-1	LIPI-1	LIPI-1 and LIPI-4	LIPI-1	LIPI-1	LIPI-1
PCR serogroup	IIa	IIa	IIa	IIa	IIa	IIb	IIa	IIa	IIa
Intact prophages (4*)*	4	3	2	1	2	1	2	1	–^*c*^
Insertion sites and length of phage region	tRNA^Arg^ (*lmot17,* 42 Kb), between ACE3HC_08995 and ACE3HC_09275 (50 Kb), tRNA^Leu^ (*lmot40,*49.3 Kb), *comK* (40.3 Kb)	tRNA^Arg^ (*lmot17*, 67.4 Kb), between ACE3HE_09440 and ACE3HE_09740 (55.7 Kb), between ACE3HE_12085 and ACE3HE_12530 (61.8 Kb)	tRNA^Arg^ (*lmot17,* 44.9 Kb), *comK* (40.5 Kb)	tRNA^Arg^ (*lmot17,* 41.8 Kb)	tRNA^Arg^ (*lmot17,* 43.2 Kb), between ACE3HL_13215 and ACE3HL_13640 (59.6 Kb)	tRNA^Arg^ (*lmot6*) (42.7 Kb)	*comK* (42.5 Kb), tRNA^Arg^ (*lmot62*, 43.5 Kb)	tRNA^Arg^ (*lmot17*, 41.7 Kb)	–^*c*^
ST^*a*^	ST155	ST9	ST504	ST16	ST173	ST87	ST14	ST37	ST403
CT^*b*^	CT1128	CT1690	CT5715	CT3732	CT3242	CT1138	CT2966	CT7559	CT40
Isolate reference	1	1	1	1	1	1	1	1	5

^*a*^ ST, sequence type (ST) according to seven-locus MLST ST according to seven-locus MLST (6).

^*b*^ CT, complex type (CT) according to 1,701 cgMLST (2).

^*c*^ no intact prophage detected.

Representative isolates were chosen from the central node of the nine cgMLST outbreak clusters described previously (1) and were thawed from glycerol stocks of the institute’s strain collection. The selected bacterial strains were cultured in 5 mL brain heart infusion broth overnight at 37°C with shaking (250 rpm), and genomic DNA was isolated from 3 mL culture with the GenElute Bacterial Genomic DNA Kit (Sigma-Aldrich) according to the instructions of the manufacturer. DNA quality was analyzed with a NanoDrop spectrophotometer (Thermo Fisher Scientific), and the DNA concentration was determined with a Qubit fluorometer (Thermo Fisher Scientific). The Rapid barcoding kit (SQK-RBK004) or Native barcoding kit 24 V14 (SQK-NBD114.24, Oxford Nanopore Technologies) was used to prepare the DNA libraries. Libraries were sequenced using FLOMIN 106D or 114 flow cells on a GridION device (Oxford Nanopore Technologies) with ont-guppy-for-gridion base call models in the super-accurate mode, a Q-score threshold of 10, and adapter trimming. The acquired long reads were filtered using Filtlong v0.2.1 (7) with standard settings, and the corresponding Illumina reads, which were taken from previous reports (1, 5), were used as an external reference. The following modifications were made: the minimal read length was set to 3,000 bp and the target bases to 290,000,000 bp, which equals a 100-fold coverage. A hybrid assembly with Unicycler v0.4.8 (8) was employed to obtain a closed genome sequence from the refined long reads and the Illumina short reads. Annotation was accomplished with the National Center for Biotechnology Information Prokaryotic Genome Annotation Pipeline v6.8 (9).

All genomes were assembled based on a 100-fold sequencing depth and had a GC content of ~38%. Genome sizes ranged from 2,911,786 bp in isolate 19-00348 with one intact prophage according to Phastest (4) to a genome size of 3,068,859 bp with four intact prophages in strain 17-01049 (Table 1). Phastest detected intact prophages in eight out of the nine genomes and indicated the tRNA^Arg^ locus (*lmot17*) as the dominant integration site, since intact prophages in this specific locus were detected in six different outbreak strains. Intact prophages inserted in this locus shared similarities with phage LP101 (NC_024387) or phage LP_HM00113468 (NC_049900) that were also found to be the most abundant prophages in serovar IIa isolates from Poland (10). Plasmids were not detected.

The genomes provided here may be helpful as reference sequences in genomic analyses, facilitating case finding and the tracing of outbreak clones to their reservoirs.

## Data Availability

The closed genome sequences and associated raw data were deposited at NCBI or the European Nucleotide Archive (ENA), respectively. All accession numbers are listed in Table 1.
